# Data-set of academic difficulties among students in western Uganda during COVID-19 induced lockdown

**DOI:** 10.1016/j.dib.2021.106851

**Published:** 2021-02-06

**Authors:** Abisha Meji M, Milon Selvam Dennison, Muhamad Mundu Mustafa

**Affiliations:** School of Engineering and Applied Sciences, Kampala International University, Western Campus, Uganda

**Keywords:** COVID-19, Lockdown, Student, Academic, Uganda, Descriptive analysis, Simple random sampling, Statistics

## Abstract

This academic research explicit the data-set of academic difficulties among different age groups of students studying in various schools, colleges or Universities during the COVID-19 induced lockdown. The western part of Uganda comprises 26 districts and the survey was conducted in those regions employing a simple random sampling technique. The dataset is descriptive and an aggregate of 405 students participated in this survey. Among that, 253 students are from rural regions, 59 students are from semi-urban regions and 93 students are from urban regions. This survey was started in April 2020 and data were collected till June 2020. A statistical run was made with the aid of SPSS version 20 software to evaluate the significance level (*P*-Value<0.05) of each question among the localities.

## Specifications Table

**Table:** 

Subject area	Education, Secondary Education
Specific subject area	Learning analytics
Type of data	Table, Excel file
How data were acquired	Survey
Data format	Raw, analyzed
Parameters for data collection	There is no parameter used for data collection. It is randomized.
Description of data collection	Data collected from the statistical population (*N* = 405) were analyzed using SPSS Version 20 software. The response rate for this questionnaire was 100% and the questions in the questionnaire were given codes with a minimum of 1 and to a maximum of 2.
Data source location	The data were obtained from the western region of Uganda which comprises 26 districts and is a landlocked region bordered to the west by the Democratic Republic of Congo (DRC), to the southwest by Rwanda, to the north and east by northern and central Uganda.
Data accessibility	Data are presented within this article and supplementary document. Repository name: Mendeley Direct URL to data: http://dx.doi.org/10.17632/wm9dg6bnkt.2

## Value of the Data

• The data presented in this article conveys the learning habits and the mental state of the students residing in the western regions of Uganda on online learning, year-end exams and career opportunities during the COVID-19 induced pandemic crisis.
• This data-set spotlights the academic difficulties facing by the students to the Government of Uganda (GoU) for taking remedial measures that could be beneficial for the students during this COVID-19 induced pandemic crisis.
• The data presented in this article will be useful for researchers to compare the learning activities of students residing in other regions of Uganda and also various developing countries during the COVID-19 induced pandemic crisis.
• The data presented in this article will be useful for researchers to compare the academic difficulties faced by the students in a pandemic situation with a normal situation.

## 1 Data Description

The everyday life of the common people around the world is highly distorted by the novel pandemic ‘COVID-19′ caused by the 2019-nCoV virus and the students around the world are not exempted from this [1], [2], [3]. Education is a gradual process of acquiring critical thinking, capabilities and expertise which helps the individual to attain personal goals and work productively for the betterment of mankind [4,5] and it is the fundamental right of every human which gives knowledge about the world and great ideas for sustaining life. Educational wealth is the one that can make a person proud and admired. Morality, character, integrity and justice are present in an educated person. Nowadays the education system has dramatically changed due to the COVID-19 induced pandemic crisis [6,7]. The school and college students all over the world are facing serious challenges due to this COVID-19 pandemic [8]. Due to the COVID-19 pandemic, the regular academic and research activities of students in Africa are distorted largely [9]. The learning habits of the students might vary during holidays when compared with the regular academic year [10,11].

A total of 405 students have participated in this survey, of that 60.2% are male students, 39.3% of students are female students and 0.5% of students have not preferred to say their gender. In this study, 52.8% of Ugandan national students have participated and the rest 47.2% are foreign students who are pursuing their studies in this locality and also the students were classified according to their age group and education level. This survey was started in April 2020 and data were collected till June 2020. The data collected for this study were analyzed by the ‘Descriptive Statistical’ method among the population (*N* = 405) using SPSS Version 20 software. The response rate for this questionnaire was 100% and the questions in the questionnaire were given codes with a minimum of 1 and to a maximum of 2. The link/copy of the survey is https://forms.gle/enMX4LxTKh1gNbsh9 and the copy of the survey has been included as a supplementary document in the data repository (Repository name: Mendeley).

To feature this dataset, total students (*N* = 405) who participated in this survey are further classified based on their localities such as rural, semi-urban, urban and the corresponding variables age, gender, nationality and education level, the details are summarized in Table 1.

**Table 1:** Demographic profile of student participants (*N* = 405).

Variables	Rural (N_1_=253) Number%	Semi-Urban (N_2_=59) Number%	Urban (N_3_=93) Number%	Total (*N* = 405) Number%
Age				
<18 Years	0 (0.0%)	0 (0.0%)	2 (2.2%)	2 (0.5%)
18 to 25 Years	169 (66.8%)	24(40.7%)	40 (43.0%)	233 (57.5%)
26 to 35 Years	64 (25.3%)	18 (30.5%)	29 (31.2%)	111(27.4%)
36 to 45 Years	15 (5.9%)	13 (22.0%)	13 (14.0%)	41(10.1%)
>45 Years	5 (2.0%)	4 (6.8%)	9 (9.7%)	18 (4.4%)
Gender				
Male	146 (57.7%)	41 (69.5%)	57 (61.3%)	244 (60.2%)
Female	106 (41.9%)	17 (28.8%)	36 (38.7%)	159(39.3%)
Prefer not to say	1 (0.4%)	1 (1.7%)	0 (0.0%)	2 (0.5%)
Nationality				
Ugandan	176 (69.6%)	14 (23.7%)	24 (25.8%)	214 (52.8%)
Non Ugandan	77 (30.4%)	45 (76.3%)	69 (74.2%)	191 (47.2%)
Education Level				
Higher Secondary School	2 (0.8%)	0 (0.0%)	3 (3.2%)	5 (1.2%)
Diploma	67 (26.5%)	4 (6.8%)	3 (3.2%)	74 (18.3%)
Under Graduate	104 (41.1%)	22 (37.3%)	35 (37.6%)	161 (39.8%)
Post Graduate	51 (20.2%)	14 (23.7%)	23 (24.7%)	88 (21.7%)
Research Scholar	29 (11.5%)	19 (32.2%)	29 (31.2%)	77 (19.0%)

As of now the COVID-19 induced lockdown made the student community stay at home for many months in Uganda [12]. The data of the academic difficulties facing by the students residing in western regions of Uganda in this COVID-19 pandemic crisis are summarized in Table 2 and the data of this survey are further summarized based on the students’ locality. The significance of each question among the students’ localities was deliberated with *P*-Value<0.05 and the corresponding data are presented in Table 3.

**Table 2:** Academic difficulties during the COVID-19 pandemic.

Item 1	Have you got your syllabus completed for this academic year?	Yes	No
		149 (36.8%)	256 (63.2%)
Item 2	Are you studying your subjects during this lockdown period?	Yes	No
		368 (90.9%)	37 (9.1%)
Item 3	Do your current studies get affected during this lockdown period?	Yes	No
		361 (89.1%)	44 (10.9%)
Item 4	If the lockdown is extended, will you submit your course works through online?	Yes	No
		364 (89.9%)	41 (10.1%)
Item 5	In what mode, you are willing to write your end semester or year-end examination?	Regular mode (Physically present after the lockdown)	Online mode
		318 (78.5%)	87 (21.5%)
Item 6	If the examination is conducted through online mode, do you have the facilities in your locality?	Yes	No
		156 (38.5%)	249 (61.5%)
Item 7	Are you aware of the open book examination system?	Yes	No
		349 (86.2%)	56 (13.8%)
Item 8	Are you learning your subjects through online mode during this lockdown period?	Yes	No
		235 (58%)	170 (42%)
Item 9	Do you feel this lockdown affected your career opportunities?	Yes	No
		352 (86.9%)	53 (13.1%)

**Table 3:** Statistical populations’ answer on the academic difficulties among students during the COVID-19 pandemic.

							95% Confidence Interval for Mean			
Item No. & Locality		N	Mean	Standard Deviation	Standard Error	Minimum	Lower Bound	Upper Bound	Maximum	*P*-Value
Item 1	Rural	253	1.7668	0.42371	0.02664	1.00	1.7143	1.8193	2.00	0.000*
	Semi-Urban	59	1.4407	0.50073	0.06519	1.00	1.3102	1.5712	2.00	
	Urban	93	1.3871	0.48973	0.05078	1.00	1.2862	1.4880	2.00	
	Total	405	1.6321	0.48283	0.02399	1.00	1.5849	1.6793	2.00	
Item 2	Rural	253	1.0632	0.24388	0.01533	1.00	1.0330	1.0934	2.00	0.023*
	Semi-Urban	59	1.1695	0.37841	0.04926	1.00	1.0709	1.2681	2.00	
	Urban	93	1.1183	0.32469	0.03367	1.00	1.0514	1.1851	2.00	
	Total	405	1.0914	0.28847	0.01433	1.00	1.0632	1.1195	2.00	
Item 3	Rural	253	1.0553	0.22909	0.01440	1.00	1.0270	1.0837	2.00	0.000*
	Semi-Urban	59	1.2203	0.41803	0.05442	1.00	1.1114	1.3293	2.00	
	Urban	93	1.1828	0.38859	0.04030	1.00	1.1028	1.2628	2.00	
	Total	405	1.1086	0.31157	0.01548	1.00	1.0782	1.1391	2.00	
Item 4	Rural	253	1.1067	0.30937	0.01945	1.00	1.0684	1.1450	2.00	0.130
	Semi-Urban	59	1.1525	0.36263	0.04721	1.00	1.0580	1.2470	2.00	
	Urban	93	1.0538	0.22677	0.02352	1.00	1.0071	1.1005	2.00	
	Total	405	1.1012	0.30201	0.01501	1.00	1.0717	1.1307	2.00	
Item 5	Rural	253	1.1383	0.34594	0.02175	1.00	1.0955	1.1812	2.00	0.000*
	Semi-Urban	59	1.2881	0.45678	0.05947	1.00	1.1691	1.4072	2.00	
	Urban	93	1.3763	0.48709	0.05051	1.00	1.2760	1.4767	2.00	
	Total	405	1.2148	0.41120	0.02043	1.00	1.1746	1.2550	2.00	
Item 6	Rural	253	1.7708	0.42118	0.02648	1.00	1.7186	1.8229	2.00	0.000*
	Semi-Urban	59	1.4407	0.50073	0.06519	1.00	1.3102	1.5712	2.00	
	Urban	93	1.3011	0.46121	0.04783	1.00	1.2061	1.3961	2.00	
	Total	405	1.6148	0.48724	0.02421	1.00	1.5672	1.6624	2.00	
Item 7	Rural	253	1.1028	0.30426	0.01913	1.00	1.0651	1.1404	2.00	0.023*
	Semi-Urban	59	1.2203	0.41803	0.05442	1.00	1.1114	1.3293	2.00	
	Urban	93	1.1828	0.38859	0.04030	1.00	1.1028	1.2628	2.00	
	Total	405	1.1383	0.34561	0.01717	1.00	1.1045	1.1720	2.00	
Item 8	Rural	253	1.5810	0.49437	0.03108	1.00	1.5198	1.6422	2.00	0.000*
	Semi-Urban	59	1.2203	0.41803	0.05442	1.00	1.1114	1.3293	2.00	
	Urban	93	1.1075	0.31146	0.03230	1.00	1.0434	1.1717	2.00	
	Total	405	1.4198	0.49413	0.02455	1.00	1.3715	1.4680	2.00	
Item 9	Rural	253	1.0711	0.25758	0.01619	1.00	1.0393	1.1030	2.00	0.000*
	Semi-Urban	59	1.2203	0.41803	0.05442	1.00	1.1114	1.3293	2.00	
	Urban	93	1.2366	0.42727	0.04431	1.00	1.1486	1.3246	2.00	
	Total	405	1.1309	0.33767	0.01678	1.00	1.0979	1.1638	2.00	

⁎ *P*-Value<0.05- Highly significant.

## 2 Experimental Design, Materials and Methods

The ‘Republic of Uganda’ is classified into four regions and the western region is one among them which comprises 26 districts. The survey was conducted in the western region of Uganda employing a simple random sampling technique. The western region of Uganda has 10 recognized Universities and its associated schools. According to the Higher Education data, more than ten thousand students are studying in those institutions.

A ‘Google Form’ was created to find the academic difficulties faced by the students during this pandemic situation. The students who are residing in the western regions of Uganda pursuing their education in higher secondary school, diploma, undergraduate, postgraduate level and research scholars were allowed to participate in this study. The non-Ugandan students who are studying in this region were also allowed without any gender bias. The questionnaire was shared among the students with the help of their school academic heads and through social networks. The design of questionnaire was made simple such that the students can respond by utilizing their smart gadgets or a personal computer from their residing places. The details of the questions and the responses are provided in the supplementary file.

## Ethics Statement

Approval to conduct this survey is obtained from the ‘Research Innovation Consultancy and Extensions (RICE)’ board of the author's University system. Informed consent was obtained from all the student participants who participated in this survey and the students who participated in this survey were voluntary and participation did not affect their grades. The questionnaire was coded to provide discretion and secrecy.

## CRediT Author Statement

**Abisha Meji M:** Methodology, Data curation, Software, Investigation, Writing-Original draft preparation; **Milon Selvam Dennison:** Conceptualization, Writing-Original draft preparation, Supervision, Validation, Writing-Reviewing and Editing; **Muhamad Mundu Mustafa:** Supervision, Writing-Reviewing and Editing.

## Declaration of Competing Interest

The authors declare that they have no known competing financial interests or personal relationships that could have appeared to influence the work reported in this article.
